# Comparison of Manual Cross-Sectional Measurements and Automatic Volumetry of the Corpus Callosum, and Their Clinical Impact: A Study on Type 1 Diabetes and Healthy Controls

**DOI:** 10.3389/fneur.2020.00027

**Published:** 2020-01-29

**Authors:** Tor-björn Claesson, Jukka Putaala, Sara Shams, Eero Salli, Daniel Gordin, Ron Liebkind, Carol Forsblom, Paula A. Summanen, Turgut Tatlisumak, Per-Henrik Groop, Juha Martola, Lena M. Thorn

**Affiliations:** ^1^Department of Radiology, Visby Regional Hospital, Visby, Sweden; ^2^Folkhälsan Institute of Genetics, Folkhälsan Research Center, Helsinki, Finland; ^3^Abdominal Center Nephrology, University of Helsinki and Helsinki University Hospital, Helsinki, Finland; ^4^Department of Radiology, Helsinki University Central Hospital, Helsinki, Finland; ^5^Department of Neurology, Helsinki University Central Hospital, Helsinki, Finland; ^6^Department of Radiology, Karolinska University Hospital, Stockholm, Sweden; ^7^Department of Clinical Neuroscience, Karolinska Institute, Stockholm, Sweden; ^8^Department of Radiology, Stanford University, Stanford, CA, United States; ^9^HUS Helsinki Medical Imaging Center, Helsinki University Central Hospital, Helsinki, Finland; ^10^Finland Research Programs Unit, Diabetes and Obesity, University of Helsinki, Helsinki, Finland; ^11^Joslin Diabetes Center, Harvard Medical School, Boston, MA, United States; ^12^Department of Ophthalmology, Helsinki University Hospital, Helsinki, Finland; ^13^Department of Clinical Neuroscience/Neurology, Institute of Neuroscience and Physiology, Sahlgrenska Academy at University of Gothenburg, Gothenburg, Sweden; ^14^Department of Neurology, Sahlgrenska University Hospital, Gothenburg, Sweden; ^15^Department of Diabetes, Central Clinical School, Monash University, Melbourne, VIC, Australia

**Keywords:** diabetes mellitus, type 1, corpus callosum, volumetry, magnetic resonance imaging, neurodegeneration

## Abstract

**Background and purpose:** Degenerative change of the corpus callosum might serve as a clinically useful surrogate marker for net pathological cerebral impact of diabetes type 1. We compared manual and automatic measurements of the corpus callosum, as well as differences in callosal cross-sectional area between subjects with type 1 diabetes and healthy controls.

**Materials and methods:** This is a cross-sectional study on 188 neurologically asymptomatic participants with type 1 diabetes and 30 healthy age- and sex-matched control subjects, recruited as part of the Finnish Diabetic Nephropathy Study. All participants underwent clinical work-up and brain MRI. Callosal area was manually measured and callosal volume quantified with FreeSurfer. The measures were normalized using manually measured mid-sagittal intracranial area and volumetric intracranial volume, respectively.

**Results:** Manual and automatic measurements correlated well (callosal area vs. volume: ρ = 0.83, *p* < 0.001 and mid-sagittal area vs. intracranial volume: ρ = 0.82, *p* < 0.001). We found no significant differences in the callosal measures between cases and controls. In type 1 diabetes, the lowest quartile of normalized callosal area was associated with higher insulin doses (*p* = 0.029) and reduced insulin sensitivity (*p* = 0.033). In addition, participants with more than two cerebral microbleeds had smaller callosal area (*p* = 0.002).

**Conclusion:** Manually measured callosal area and automatically segmented are interchangeable. The association seen between callosal size with cerebral microbleeds and insulin resistance is indicative of small vessel disease pathology in diabetes type 1.

## Introduction

Corpus Callosum (CC) is the largest commissure of the brain, responsible for much of inter-hemispheric communication (1). Decrease in cross-sectional sagittal area of the CC (CCA) has been used as a surrogate for cerebral atrophy, based on the observation that CCA is proportional to the number of axons passing through the CC (1, 2), and that callosal axon density does not decrease with callosal size (1). Furthermore, as an advantage, CCA does not decrease, or decreases only slightly (3), with normal aging, up until at least the 7:th-8:th decade (4, 5).

The number of axons passing through the CC is decreased by at least two neuro-degenerative processes: cortical atrophy and neuronal cell death, which cause axonal loss by Wallerian degeneration, and myelin break-down with central atrophy as observed in cerebral small vessel disease (6, 7). To get a measure of callosal shrinkage, and to correct for individual differences in brain size, CCA can be normalized to mid-sagittal cross-sectional area (MISS). MISS functions as a surrogate for maximally attained brain size (4), and this normalized measure does not differ between the sexes (4).

CCA and MISS can be easily measured in MRI-scans using the tools available on standard PACS workstations (8). CCA/MISS could be a useful method to rapidly estimate pathological CNS effects of disease, possibly as an alternative to volumetric measurements, that yield the corresponding CC volume (CCV) and estimated total intracranial volume (eTIV). The correlation between CCA and CCV or MISS and eTIV has been studied in a smaller material. The agreement between measurements made by junior physicians and experienced neuro-radiologists was found to be excellent, which means that CCA can be easily measured in routine clinical practice (9). Not all imaging centers are equipped to routinely perform volumetry, and simple manual measurements could thus prove valuable.

We recently observed that neurologically healthy participants with type 1 diabetes (T1D) had more frequent manifestations of cerebral small vessel disease on brain MRI compared with healthy controls (10). Knowing individuals with T1D show clear signs of early vascular aging (11), our question was whether premature pathology can be found also in central structures of the brain.

We hypothesized that individuals with T1D might have smaller CC size than healthy control subjects and that the signs of small vessel disease might correlate with CC size.

We first aimed to assess the correlation between CCA/MISS and CCV/eTIV. Second, we studied, whether CCA/MISS differs between those with T1D and healthy control subjects, and whether it is associated with diabetes related traits and/or MRI-signs of cerebral small vessel disease.

## Materials and Methods

### Study Population

This study is part of the nationwide, prospective Finnish Diabetic Nephropathy Study. For this cross-sectional sub study, 191 participants with T1D were examined between 2011 and 2017, and 30 healthy age- and sex-matched control subjects in 2017. The study design has been described elsewhere in detail (10, 12). T1D was defined as disease onset before 40 years and insulin treatment initiated within one year of diagnosis. Inclusion criterion was T1D without history of cerebrovascular disease, and exclusion criterion the presence of end-stage renal disease. For this sub study, we excluded one participant with T1D and history of brain surgery, and two participants with MS, since the disease influences the CC (13). The controls were non-diabetic age- and sex-matched volunteers, without first degree relatives having diabetes. The Ethics committee of Helsinki and Uusimaa Health District approved our study, and we adhered to the Declaration of Helsinki.

### Clinical Data

All participants underwent a clinical work-up at the Finnish Diabetic Nephropathy Study center at the Helsinki University Hospital. This included a thorough review of the patient's medical history, anthropometric measurements, as well as questionnaires about lifestyle factors such as smoking. Blood samples were drawn and analyzed for HbA_1c_, lipids and lipoproteins, and creatinine. Estimated glomerular filtration rate was calculated with the CKD-EPI formula (14). Urinary albumin excretion rate was measured from a 24-h urine collection. Insulin sensitivity was calculated with the formula for the estimated glucose disposal rate (eGDR) (12, 15).

Diabetic complications were registered, and included coronary heart disease, retinal photocoagulation, and albuminuria. None of the study participants had a history of peripheral arterial disease.

### Imaging Protocols and Image Analysis

All participants underwent a brain MRI examination within 1 year from the clinical work-up at the Helsinki Medical Imaging Center, Helsinki University Hospital, using one 3T scanner (Achieva, Philips, Best, The Netherlands), which was not upgraded, but decommissioned before more control participants could be recruited. MRI sequences used were part of a standard protocol and include: T1, T2, FLAIR, T1 MPRAGE, DWI, SWI, T2^*^, and MRA TOF sequences (Supplemental Table 1).

Images were assessed for the following signs of cerebral small-vessel disease (10): the presence of cerebral micro-bleeds, cortical superficial siderosis, white-matter hyper-intensities (Fazekas scale used, with category one considered to be a significant burden), or lacunes (16, 17). None of the participants had cortical superficial siderosis.

**Figure 1:**
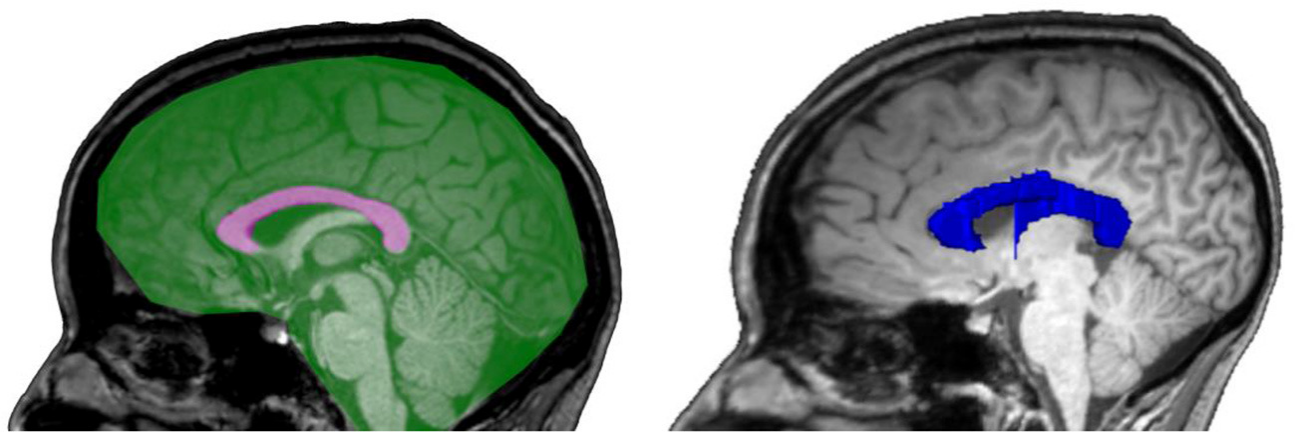
Schematic illustrations of manually measured 2D areas of CCA (pink), MISS (green) and an example of a FreeSurfer-segmentation of CCV (blue).

For manual measurement of CCA and MISS the T1 MPRAGE data set was formatted as straight sagittal slices and inspected in a mid-sagittal slice, defined by the central axis of the aqueduct. CCA and MISS were measured by outlining their contours using the standard area tracing tool of the Agfa Impax workstation, see Figure 1. Image analysis was done by a senior neuro-radiologist (J.M.) with > 10 years of experience, who was blinded to the clinical parameters and the results of volumetry. CCA and MISS were measured three times each at the same occasion, to assess internal validity of the area measurement.

We normalized CCA to MISS to correct for differences in maximally attained brain size, and to get a measure of reduction of CCA.

The FreeSurfer software, version 6.0, was used for volumetry using the T1 MPRAGE sequence, http://surfer.nmr.mgh.harvard.edu/. FreeSurfer measures CCV by finding the largest white matter connection between the hemispheres, divides it into a number of equally long segments, which are then extruded to yield volumes. The number of segments and extrusion width is user configurable. Intracranial volume was estimated using atlas normalization (18). The FreeSurfer rater was masked to all clinical parameters, radiological findings, and the results of manual neuroradiological measurements.

### Statistical Analysis

All continuous variables were tested for normality of distribution. Normally distributed variables were tested in group comparisons with Students *t*-test or ANOVA (for multiple groups) and results presented as mean ± standard deviation. Variables with non-normal distribution were tested for group differences with Mann–Whitney's test or Kruskal–Wallis (for multiple groups) and results presented as medians with inter-quartile range.

One-way intra-class correlation coefficient test was performed for the 3 separate measurements of each participant's CCA and MISS, for intra-rater reliability. The CCA and MISS reported are the means of the three separate measurements. Correlation between manual CC measurements and automated volumetric measurements, as well as correlation between normalized CCA/MISS, CCV/eTIV, and a compound CCA/eTIV were examined with Pearson's or Spearman's (when not normally distributed) correlation tests.

Differences in clinical parameters in the T1D group were assessed between group quartiles based on CCA/MISS. We also grouped cases based on the number of cerebral microbleeds (zero, one to two, more than two).

We performed logistic regression on the individuals with T1D. Outcomes were belonging to the quartile with the smallest CCA_mean_/MISS_mean_ vs. any other quartile. Independent variables were sex, age, insulin dose/kg, eGDR, and number of cerebral microbleeds. Waist-to-hip ratio was included in a separate model instead of eGDR due to collinearity. Results are expressed as OR with 95% CI.

Statistical analysis was done using R (http://www.r-project.org) version 3.4.3 “Kite-Eating Tree” and *p* < 0.05 was set as the threshold for statistical significance.

## Results

### Callosal Measurements

**Figure 2:**
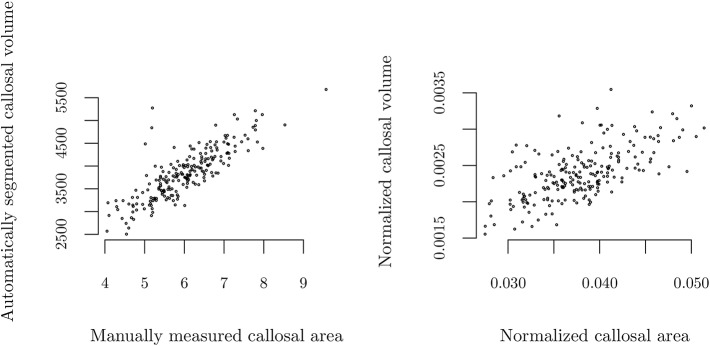
Correlation of manually measured callosal area and automated measurements of callosal volume using FreeSurfer. Absolute values **(left)**, ρ = 0.83 (*p* < 0.001), and normalized to mid-sagittal area or estimated total intracranial volume, respectively **(right)**, ρ = 0.61 (*p* < 0.001).

CCA and MISS measurements had an intra-class correlation coefficient of 0.989 (95% CI: 0.987–0.992) and 0.996 (95% CI: 0.995–0.997), respectively. As depicted in Figure 2, the correlation between CCA and CCV was ρ = 0.83 (*p* < 0.001). MISS showed a correlation with eTIV ρ = 0.82 (*p* < 0.001). The correlation between normalized CCA/MISS and CCV/eTIV was ρ = 0.61 (*p* < 0.001).

When manually inspecting the FreeSurfer-segmented volumes we found at least part of the fornix to be included in the vast majority of segmentations and that the segmented area often bleeds outside of the anatomical boarders of the callosum. We did not correct for this tendency of FreeSurfer to segment too large CCV.

There were no significant differences between cases and controls in callosal measurements. CCA/MISS was 0.037 (0.034–0.041) and 0.039 (0.036–0.041) for the T1D and controls groups respectively, *p* = 0.11.

### Clinical Parameters

**Figure 3:**
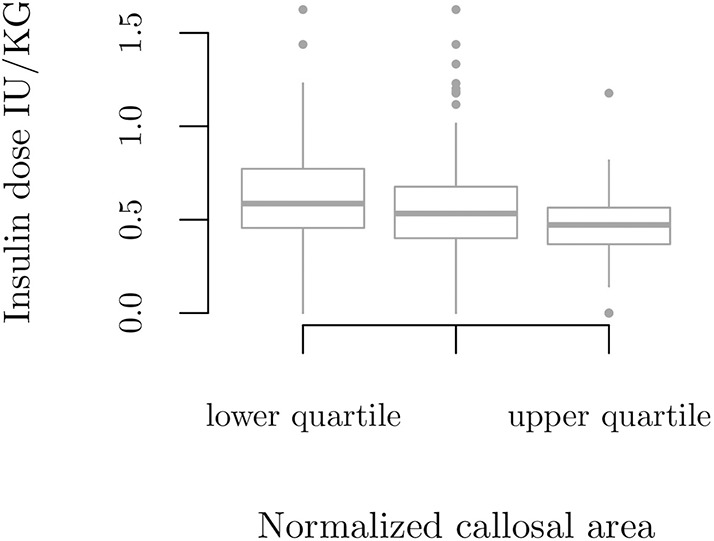
Insulin dose per body weight according to quartile groups based on callosal area normalized by mid-sagittal intracranial area, *p* = 0.035.

We grouped the participants with T1D based on quartiles of CCA/MISS, for which clinical and radiological characteristics appear in Table 1. Those in the lowest quartile of CCA/MISS had higher daily insulin doses (*p* = 0.035), Figure 3, and those in the highest quartile were more insulin sensitive (*p* = 0.009).

**Table 1:** Characteristics of participants according to quartiles of CCA/MISS: Data are percentage, mean ± standard deviation if normally distributed, or median (inter-quartile range).

	**Lower quartile, *N* = 47**	**Middle quartiles, *N* = 94**	**Upper quartile, *N* = 47**	**Controls (*N* = 30)**	p^*^	p^**^
CCA/MISS	(0.0275, 0.0343)	(0.0343, 0.0406)	(0.0406, 0.0514)	–	0.0389 (0.0361–0.0411)	0.040
Age (years)	39.9 (31.6–44.0)	40.4 (34.4–45.1)	38.9 (32.6–45.6)	38.4 (32.2–42.9)	0.534	0.280
Age at diabetes onset (years)	11.4 (5.6–17.3)	15.2 (9.0–24.5)	14.0 (9.5–24.0)	NA (NA–NA)	0.091	–
Diabetes duration (years)	22.8 (18.0–35.3)	21.4 (18.3–29.5)	21.7 (18.4–28.4)	NA (NA–NA)	0.589	–
Male gender (%)	49	51	36	43	0.312	–
HbA_1c_ (mmol/mol)	65 (57–73)	66 (59–76)	64 (56–69)	33 (31–34)	0.201	0.000
Insulin dose (IU/kg)	0.59 (0.46–0.77)	0.53 (0.40–0.70)	0.47 (0.37–0.56)	NA (NA– NA)	0.029	–
eGDR (mg/kg/min)	7.79 (6.02–9.41)	7.14 (5.39–8.56)	8.46 (6.56–9.17)	10.29 (9.43–10.98)	0.033	0.000
History of smoking (%)	30	39	23	40	0.270	0.951
Established coronary heart disease (%)	0	1	0	0	0.392	0.572
Retinal photocoagulation (%)	23	23	17	0	0.840	0.004
Urinary albumin excretion (mg/24h)	13 (11–18)	14 (10–20)	12 (9–16)	13 (11–16)	0.379	0.229
Glomerular filtration rate [ml/(min^*^1,73^*^m^2^)]	108 (94–117)	108 (96–113)	108 (100–115)	101 (86–110)	0.210	0.115
Warfarin use (%)	0	1	0	0	0.392	0.572
Aspirin treatment (%)	6	7	11	0	0.851	0.125
Antihypertensive medication (%)	38	40	21	0	0.110	0.000
Body mass index (kg/m^2^)	25.9 (23.9–28.9)	26.5 (24.1–29.8)	25.9 (23.4–28.6)	24.4 (22.2–25.7)	0.643	0.002
Waist to hip ratio	0.87 (0.80–0.95)	0.89 (0.84–0.96)	0.85 (0.80–0.91)	0.85 (0.80–0.92)	0.033	0.014
Systolic blood pressure (mmHg)	130 ± 15	130 ± 14	129 ± 16	121 ± 11	0.646	0.001
Diastolic blood pressure (mmHg)	76 ± 9	77 ± 8	75 ± 9	78 ± 8	0.388	0.655
Total cholesterol (mmol/l)	4.39 (3.88–5.00)	4.48 (4.06–5.00)	4.40 (4.10–4.79)	4.60 (4.20–5.40)	0.489	0.491
LDL cholesterol (mmol/l)	2.27 (1.73–2.82)	2.44 (2.06–3.01)	2.37 (2.08–2.90)	2.62 (2.33–3.28)	0.346	0.108
HDL cholesterol (mmol/l)	1.59 (1.38–1.80)	1.40 (1.20–1.80)	1.52 (1.27–1.84)	1.46 (1.28–1.66)	0.339	0.854
Triglycerides (mmol/l)	0.92 (0.77–1.27)	1.00 (0.67–1.56)	0.81 (0.64–1.04)	0.84 (0.70–1.24)	0.202	0.244
Lacunar infarctions (%)	4	2	0	0	0.566	0.422
Cerebral microbleeds (%)	28	20	26	3	0.758	0.029
Signs of cerebral small vessel disease (%)	38	33	36	10	0.927	0.014

p* *–between patient quartiles*,

p** *–between controls and middle patient quartiles. Clinical parameters in participants with type 1 diabetes split into quartiles based on normalized mid-sagittal callosal area. Control subjects are included and compared against the mid quartiles*.

**Figure 4:**
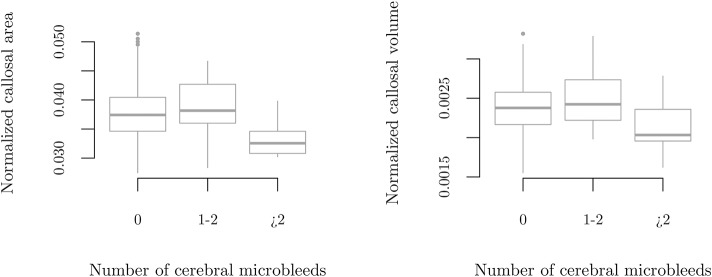
Callosal size by number of cerebral microbleeds in participants with type 1 diabetes. Manually measured callosal area normalized by mid-sagittal intracranial area (left), p = 0.002, and callosal volume normalized by estimated total intracranial volume as measured by FreeSurfer (right), p = 0.018.

There were no significant differences in the markers of cerebral small vessel disease between the quartiles of CCA/MISS. There were, however, a number of participants with several cerebral microbleeds. When we disregarded participants without any microbleeds, the mean number of microbleeds by CCA/MISS were: lowest quartile 5 (2–18), middle quartiles 1 (1–2), and highest quartile 1 (1–1), *p* = 0.003. With the T1D group subdivided based on the number of cerebral microbleeds (zero [*N* = 144], one to two [*N* = 32], more than two [*N* = 12]), CCA/MISS was 0.037 (0.035–0.040) vs. 0.038 (0.036–0.043) vs. 0.033 (0.031–0.035), *p* = 0.002, Figure 4.

In logistic regression analysis an independent association was found between lowest quartile of CCA/MISS and higher insulin dose: OR per 1 IU/kg 5.38 (95 % CI 1.38–21.04), *p* = 0.016, as well as with the presence of more than two cerebral microbleeds vs. zero: OR 12.00 (2.85–50.65), *p* = 0.001.

## Discussion

Manual and automatic measurements of the CC, MISS and eTIV, are correlated. We observed significant differences in clinical characteristics between participants grouped by quartiles of normalized CCA—those with the smallest CCA had higher daily insulin doses and those with the largest CCA had the highest insulin sensitivity. When split by number of cerebral microbleeds, those with more than two cerebral microbleeds had smaller normalized CCA. Interestingly, we found no significant CC differences between cases and controls.

The correlations between CCA to CCV and MISS to eTIV were ρ = 0.8. Previous studies comparing manually segmented volumes vs. FreeSurfer have shown similar correlations of *r* = 0.74 (19) and *r* = 0.82 (20) for hippocampal volume. Segmentation of CCV using FreeSurfer has been evaluated by comparison to manually corrected CCV-segmentation, and found to perform very well (21). This was however using a previous version of FreeSurfer. With version 6.0, we find that FreeSurfer-segmented CCV tends to be too large compared to CCA outlined by an experienced neuro-radiologist. This is because of frequent inclusion of a variably large part of the fornix as well as bleeds of the segmented area outside the callosum. The variable error thus introduced causes the correlation of CCA vs. CCV to suffer. FreeSurfer version 6.0 was however found to correlate well with manual measurement using the longitudinal stream by Platten et al. (9). The correlation was worse using the cross-sectional stream as is the case in the present study.

One potential advantage of CCV over CCA is the ability of FreeSurfer to deal with a misaligned or skewed mid-line. It should however be possible to overcome any problems with misaligned mid-lines even in clinical practice given the almost universal availability of 3D-imaging and multi planar reconstruction. The biggest contributor to the discrepancy in the present study between manual and FreeSurfer based callosal measurements is the above mentioned tendency to over-segmentation.

The correlation for CCA/MISS and CCV/eTIV was weaker, ρ = 0.6, which may be because the planar measurements did not encompass skull width. Because it does take skull width into account, the volumetric eTIV (18), is a conceptually better estimate of total intracranial volume than MISS. CCA/eTIV could be an attractive normalized measure, although it did not provide any stronger correlation with the clinical parameters than CCA/MISS or CCV/eTIV, or differ between cases and controls in our cohort (data not shown).

In our study, the intraclass correlations of the CCA and MISS measurements were excellent. Repeated measurements in the same slice using standard PACS area-tracing tools gave highly uniform results suggesting there is no advantage to multiple averaged measurements over single measurements. CCA measurements have been found to be highly repeatable regardless of operator experience (9).

The mean CCA for our control participants was 6.16 cm^2^, which is close to those reported by Mitchell et al. (22) and Sullivan et al. (4).

We found an association between CC atrophy and cerebral microbleeds. This finding may indicate that the underlying small vessel disease in individuals with T1D, to the extent it is causing the microbleeds, is associated with further brain degeneration. More than 5 micro-bleeds indicate higher risk of future cerebral hemorrhage and might influence treatment choices (23).

Callosal atrophy has been observed in diseases with white matter lesions secondary to cerebral small vessel disease, such as vascular dementia and CADASIL (24). Future studies will tell whether the changes observed in our study will translate into cognitive impairment.

We found higher insulin dose, suggesting insulin resistance, to be associated with decreased CCA. This is supported by higher eGDR, indicating higher insulin sensitivity, being associated with larger CCA. Interestingly, insulin resistance and/or hyperinsulinemia has been implicated in the pathogenesis of Alzheimer's disease (25). In T1D, on the other hand, CNS involvement such as cognitive impairment has been associated with white matter atrophy (26) and retinopathy (27, 28).

In type 2 diabetes, a non-significant trend toward atrophy of the CC has been noted compared to healthy controls (29). CC atrophy has also been observed in MS, where it associates with decreasing cognitive ability, disease duration, and other measures of disease severity (13). Similarly, in people with Alzheimer's and Parkinson's disease CC atrophy is associated with more severe disease stages (30–32). This is in line with our results, as no significant difference was shown in CC size between healthy controls and neurologically asymptomatic individuals with T1D. The lack of difference in our cohort may, thus, indicate that the CC is less affected at the early disease stages in young individuals.

The strengths of our study include a well-defined and thoroughly clinically characterized cohort. Our measurements were done on the same sequences with imaging performed on the same scanner. The mid-sagittal area for each scan was defined by a technician, masked to the study, which further limits bias of measurements. Limitations include the rather low number of control participants, which limits the ability of the study to detect small group differences and increases the risk of type 2 errors, as well as the cross-sectional design of the study.

## Conclusion

Manual cross-sectional and automatic volume measurements of the CC correlate well. CC size does not differ between T1D and control groups. However, multiple cerebral micro-bleeds, eGDR (as a marker of lower insulin sensitivity), and higher insulin doses were associated with CC size in T1D. This indicates a silent role of small vessel disease in early T1D that does impact brain health. Future studies will focus on T1D and brain health longitudinally.

## Data Availability Statement

The datasets generated for this study will not be made publicly available as they are covered by patient confidentiality.

## Ethics Statement

The studies involving human participants were reviewed and approved by The Ethics committee of Helsinki and Uusimaa Health District. The patients/participants provided their written informed consent to participate in this study.

## Author Contributions

TC, LT, SS, ES, DG, RL, CF, PS, TT, JP, JM, and P-HG contributed to the study design, acquisition of data, as well as the interpretation of data. TC and LT had the main responsibility for analyzing the data and writing the first draft of the paper. SS, ES, DG, RL, CF, PS, TT, JP, JM, and P-HG critically revised the manuscript. P-HG, is the guarantor of this work and, as such, had full access to all the data in the study, and takes responsibility for the integrity of the data and the accuracy of the data analysis. All authors have reviewed and accepted the submitted version of this manuscript.

### Conflict of Interest

PS has received lecture honoraria from Bayer and Santen. TT is an advisory board member of Boehringer Ingelheim, Bayer, Pfizer, and Lumosa Therapeutics, and has received speaker honoraria from the University of Donau (Austria). P-HG has received lecture honoraria from AstraZeneca, Boehringer Ingelheim, Eli Lilly, Elo Water, Genzyme, Medscape, MSD, Novartis, Novo Nordisk, and Sanofi, and he is an advisory board member of AbbVie, Boehringer Ingelheim, Eli Lilly, Janssen, Medscape, MSD, Novartis, Novo Nordisk, and Sanofi. JM has received lecture honoraria from Santen. The remaining authors declare that the research was conducted in the absence of any commercial or financial relationships that could be construed as a potential conflict of interest.

## Abbreviations

CC: Corpus Callosum
CCA: Corpus Callosum Area
MISS: Mid-sagittal cross-sectional area
CCV: Corpus Callosum Volume
eTIV: Estimated Total Intracranial Volume
T1D: Type 1 diabetes.
