# Supplementary material for: Comparison of Manual Cross-Sectional Measurements and Automatic Volumetry of the Corpus Callosum, and Their Clinical Impact: A Study on Type 1 Diabetes and Healthy Controls
Source: Front Neurol. 2020 Jan 29;11:27. doi: 10.3389/fneur.2020.00027 (PMC7000520; doi:10.3389/fneur.2020.00027)
Supplement: Supplementary file 1 [file Data_Sheet_1.PDF]

Contents

1 Supplemental materials **1**

1 Supplemental materials

Table 1: Supplemental table 1, MRI sequence parameters

Sequence	TR	TE	Flip angle	Echos	Voxel size
T1 MPRAGE	shortest	4.6 ms	8°	1	0.88x0.88x0.88 mm
3D TOF	25 ms	3.5 ms	20°	1	0.28x0.54x0.50 mm
3D SSH	shortest	46 ms	90°	1	2.00x2.00 mm
T1 IR TSE	2000 ms	10 ms	(refocus 120°)	1	0.98x1.22 mm
T2 FFE	shortest	16 ms	18	1	0.90x1.12 mm
T2 FLAIR	11000 ms	125 ms	(refocus 120°)	1	0.65x0.87 mm
T2 TSE	4000 ms	80 ms	90°	1	0.60x0.75 mm
VEN BOLD	shortest	shortest	10°	1	1.00x0.99x0.50 mm
